# Recent advances in dissolving microneedles for breast cancer immunotherapy: local delivery and tumor microenvironment remodeling

**DOI:** 10.3389/fimmu.2026.1831221

**Published:** 2026-05-15

**Authors:** Yingcui Chen, Li Song, Yingze Zhu, Zhuoqi Zhang, Yige Lu, Xinyue Wang, Hui Pang

**Affiliations:** 1 The Fourth Department of Medical Oncology, Harbin Medical University Cancer Hospital, Harbin, Heilongjiang, China; 2 Department of Oncology, Jiamusi Tumor Hospital, Jiamusi, Heilongjiang, China

**Keywords:** breast cancer, dissolving microneedles, immunotherapy, transdermal immunodelivery, tumor immune microenvironment

## Abstract

Breast cancer is generally characterized by limited immunogenicity. Although immune checkpoint inhibitors (ICIs) provide survival benefits in a subset of patients with triple-negative breast cancer (TNBC), overall response rates remain limited and are accompanied by pronounced therapeutic heterogeneity and systemic immune-related adverse events. The immunosuppressive tumor immune microenvironment (TIME), characterized by restricted antigen presentation, limited effector T-cell infiltration, and enrichment of immunosuppressive cell populations, represents a key barrier to durable immunotherapy benefit. Dissolving microneedles (DMNs) represent a minimally invasive transdermal delivery system that bypasses the stratum corneum barrier. They enable the delivery of antigens and immunomodulatory molecules into the epidermis and superficial dermis enriched in antigen-presenting cells (APCs), thereby facilitating efficient peripheral immune priming while reducing systemic exposure. This review summarizes the material composition and microstructural design of DMNs, their transdermal immunodelivery characteristics, and recent advances in breast cancer immunotherapy. It further highlights the mechanisms by which DMNs mediate peripheral immune activation and remodel the TIME, as well as their potential in combination with other immunotherapeutic strategies. Overall, DMNs represent a promising strategy that may improve the efficacy and safety of breast cancer immunotherapy by enabling localized delivery, efficient peripheral activation, and coordinated microenvironment remodeling.

## Graphical abstract

**Figure:**
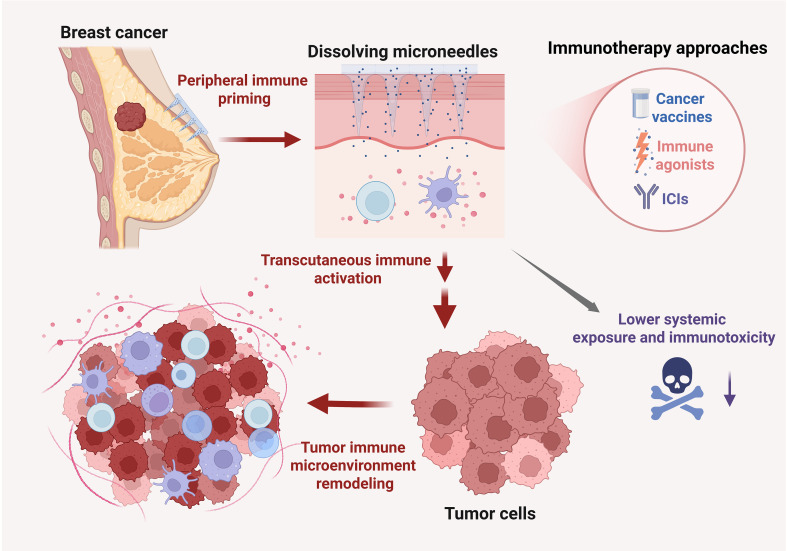

## 1 Introduction

Breast cancer is one of the most commonly diagnosed malignancies among women worldwide and remains a leading cause of cancer-related mortality. According to global cancer statistics, approximately 2.3 million new cases of breast cancer were diagnosed worldwide in 2022, accounting for more than 11% of all newly diagnosed cancers, with an estimated 660,000 associated deaths. Notably, the incidence of breast cancer has continued to rise over the past decade in most countries (1, 2). Despite advances in early screening, molecular subtyping, and multimodal treatment strategies that have modestly improved patient outcomes, breast cancer continues to face substantial challenges in terms of recurrence, metastasis, and the long-term durability of therapeutic efficacy. These epidemiological trends underscore the urgent need for more effective and durable therapeutic strategies.

From a therapeutic perspective, the clinical management of breast cancer has progressively evolved from traditional strategies centered on surgery, chemotherapy, and radiotherapy toward more integrated precision treatment approaches that combine multiple modalities, including human epidermal growth factor receptor 2 (HER2)–targeted therapy and antibody–drug conjugates (ADCs). However, the therapeutic efficacy of these strategies varies markedly across molecular subtypes and is frequently constrained by treatment resistance, systemic toxicity, and a narrow therapeutic window that limits dose optimization and long-term efficacy. In recent years, immunotherapy, particularly immune checkpoint inhibitors (ICIs), has achieved breakthrough success in multiple solid tumors, providing new therapeutic opportunities for breast cancer. Nevertheless, in contrast to melanoma or non-small cell lung cancer, breast cancer is generally characterized by lower immunogenicity and limited responsiveness to immunotherapy. Accumulating evidence indicates that most breast cancers, especially hormone receptor–positive subtypes, exhibit impaired antigen presentation, a paucity of tumor-infiltrating lymphocytes (TILs), and enrichment of immunosuppressive cell populations, including myeloid-derived suppressor cells (MDSCs) and regulatory T cells (Tregs), collectively contributing to the formation of an immunosuppressive tumor microenvironment (TME) (3, 4). Even in TNBC, which displays relatively higher immunogenicity, ICIs combined with chemotherapy have demonstrated clinical benefit in only a subset of patients, while pronounced response heterogeneity and systemic immune-related adverse events continue to substantially limit their broader application (5, 6). Consequently, achieving effective and controllable local immune activation while reducing systemic toxicity and remodeling the immunosuppressive TME has emerged as a critical unmet need and a key direction for improving the efficacy and safety of breast cancer immunotherapy.

Against this backdrop, increasing attention has been directed toward regulating the spatial context of immune activation by reconstructing the initiation of immune responses through localized delivery strategies. The skin is the largest immune organ in the human body and is enriched with dendritic cells, Langerhans cells, and diverse innate immune sensing elements, which together provide a unique biological foundation for the recognition and amplification of exogenous antigens and immunomodulatory signals (7–9). Transdermal delivery systems that exploit the immunological advantages of the skin have therefore emerged as an important research direction in cancer immunotherapy because they enable efficient and safe immune modulation at peripheral immune sites. Microneedles (MNs), defined as micron-scale needle arrays capable of penetrating the stratum corneum with minimal invasiveness, represent a novel drug delivery platform that bridges conventional injections and passive transdermal diffusion. By creating transient microchannels in the superficial layers of the skin, MNs allow the precise delivery of therapeutics into the epidermis and superficial dermis enriched in antigen-presenting cells under minimally painful conditions (10, 11). With advances in materials science and microfabrication technologies, microneedle systems have diversified into multiple formats and demonstrated broad application potential in vaccination, localized immune modulation, and cancer therapy (12, 13). Among the various types of microneedle systems, DMNs have attracted particular attention due to their unique structural and delivery advantages. DMNs are fabricated from biodegradable materials and dissolve within the skin following insertion to release their payloads, offering high drug-loading capacity, favorable biocompatibility, and improved safety by avoiding needle residue.

Consequently, they are considered one of the microneedle platforms with high translational potential (14, 15). Based on these attributes, the application of DMNs in breast cancer immunotherapy, particularly in triple-negative breast cancer (TNBC) -related preclinical models, has expanded in recent years, with multiple studies demonstrating more favorable immune activation profiles and antitumor efficacy compared with systemic administration (16–18). This Review summarizes recent advances in DMNs for breast cancer immunotherapy, focusing on localized delivery strategies, tumor microenvironment remodeling mechanisms, and translational potential. It also offers an integrated perspective on how localized immunodelivery may influence immune regulation and therapeutic responses in breast cancer.

## 2 Breast cancer immunological basis

### 2.1 Current status of immunotherapy

Although ICIs have reshaped the therapeutic landscape of multiple highly immunogenic malignancies, breast cancer as a whole is still regarded as a prototypical immunologically “cold” tumor, with substantially lower sensitivity to systemic immunotherapy than immunoresponsive tumors such as melanoma or non-small cell lung cancer (19). Across the breast cancer spectrum, TNBC is currently the only major subtype that has demonstrated a clear clinical benefit from ICIs, which is generally attributed to its relatively higher mutational burden, more abundant TILs, and more active inflammation-associated signaling. The phase III KEYNOTE-355 trial showed that, in patients with metastatic TNBC and a PD-L1 combined positive score (CPS) ≥10, pembrolizumab in combination with chemotherapy significantly improved progression-free survival (PFS) and overall survival (OS). This established the regimen as a first-line treatment option for PD-L1–positive disease (20). Similarly, the IMpassion130 trial confirmed the survival benefit of atezolizumab combined with nab-paclitaxel in the same patient population, providing pivotal evidence supporting the incorporation of immunotherapy into first-line treatment for TNBC (21). In parallel, the success of the KEYNOTE-522 trial moved immunotherapy into the neoadjuvant setting for high-risk early-stage TNBC, in which neoadjuvant pembrolizumab plus chemotherapy significantly increased the rate of pathologic complete response (pCR) and resulted in a clinically meaningful improvement in event-free survival (EFS) during long-term follow-up (22). Together, these clinical trials highlight the expanding role of ICIs across different treatment settings in TNBC, including metastatic and early-stage disease, although their clinical benefits remain heterogeneous across patients.

Despite these promising advances, even within the relatively immunogenic TNBC subtype, the overall response rate to ICIs remains limited (23, 24). This limited response is largely attributed to multiple underlying biological factors. Accumulating evidence indicates that the response of breast cancer to ICIs is not determined by a single factor but is jointly regulated by multiple biological features, including tumor mutational burden (TMB), levels of TILs, and PD-L1 expression status, with high TMB and abundant TILs generally associated with enhanced antigen immunogenicity and improved responsiveness to immunotherapy. In addition, molecular alterations associated with immune evasion, such as loss of β2-microglobulin (B2M) or human leukocyte antigen A (HLA-A), as well as JAK1/2 mutations, can impair tumor sensitivity to immune checkpoint blockade by disrupting antigen presentation integrity or interferon signaling, respectively. However, the clinical utility of these alterations remains largely confined to mechanistic studies and exploratory analyses (25, 26). PD-L1 expression has been widely used for clinical patient stratification. In breast cancer, PD-L1 expression is characterized by pronounced spatial heterogeneity. These inconsistencies in detection platforms and scoring criteria further limit its stability and generalizability as a predictive biomarker (27, 28). Meanwhile, mechanisms of resistance to immunotherapy can arise from both tumor-intrinsic and tumor-extrinsic factors. These include defects in antigen presentation, activation of alternative immune checkpoint pathways, and the sustained influence of immunosuppressive cell populations, such as Tregs and MDSCs, as well as metabolic or hypoxic stress (29–31). Collectively, these factors shape a complex and dynamic immune evasion network in breast cancer, rendering it difficult to consistently establish effective antitumor immune responses through systemic administration alone. Furthermore, from a functional immunological perspective, the effector immune state reflected by TILs is commonly accompanied by interferon-γ–related signaling pathways, which contribute to the amplification of antitumor immune responses following immune checkpoint blockade (32, 33). Notably, although multiple studies suggest an association between high levels of TILs and immunotherapy benefit, limitations related to detection methods, threshold definitions, and spatial heterogeneity currently preclude their use as a standardized predictive biomarker in routine clinical practice, highlighting the limitations of relying solely on these biomarkers to guide immunotherapy in breast cancer.

By contrast, hormone receptor–positive (HR^+^)/HER2^-^ and HER2^+^ breast cancers generally exhibit more pronounced immunosuppressive features, including low levels of TILs, inefficient antigen presentation, and enrichment of immunosuppressive cell populations. Accordingly, whether administered as monotherapy or in combination with chemotherapy, anti-HER2 therapy, or endocrine therapy, ICIs have shown relatively limited clinical benefit in these subtypes. Although multiple clinical trials are currently exploring combination strategies involving ICIs with CDK4/6 inhibitors, ADCs, or immunostimulatory adjuvants, the available evidence remains largely exploratory and is insufficient to support their widespread incorporation into routine clinical practice at present (34). In addition, systemic immunotherapy is inevitably associated with immune-related adverse events (irAEs), which can affect multiple organ systems, including the skin, endocrine organs, liver, and lungs, and are of particular clinical relevance in breast cancer populations with relatively long expected survival durations. Therefore, enhancing antitumor immune efficacy while reducing systemic exposure and toxicity has become one of the central challenges in breast cancer immunotherapy. This limitation highlights the need for localized delivery strategies to achieve effective immune activation while minimizing systemic toxicity. Addressing this challenge requires strategies that improve the local TME, enhance antigen presentation, and increase immune cell infiltration, while simultaneously reducing systemic toxicity through more precise and localized delivery approaches (35, 36). In this context, achieving fine control over both the intensity and spatial localization of immune activation while minimizing systemic exposure has emerged as a key issue for advancing breast cancer immunotherapy.

### 2.2 Tumor immune microenvironment

The TME of breast cancer largely determines the magnitude and stability of responses to immunotherapy. Tumor cells can actively establish an immunosuppressive tumor immune microenvironment (TIME) by modulating immune-related signaling pathways, restricting antigen recognition, and inducing T cell exhaustion. These processes impair the initiation and maintenance of antitumor immune responses (37). In most breast cancers, particularly the HR^+^/HER2^-^ subtype, this immunosuppressive state is characterized by inefficient antigen presentation, impaired maturation of dendritic cells (DCs), and limited infiltration of effector T cells into the tumor core (38, 39). Meanwhile, sustained accumulation of immunosuppressive cell populations, including MDSCs, Tregs, and M2-polarized tumor-associated macrophages (TAMs), suppresses T cell function and attenuates local immune activation through multiple mechanisms, such as TGF-β, IL-10, IDO activity, and lactate-driven metabolic reprogramming (40). In addition, stromal remodeling mediated by CAFs and aberrant tumor vasculature restrict immune cell trafficking into the tumor core. It also promotes the establishment and maintenance of an immunosuppressive microenvironment by altering local metabolic and oxygenation conditions. At the subtype level, marked differences in immune activity are observed across breast cancer TMEs. TNBC generally exhibits higher immunogenicity but displays pronounced intratumoral heterogeneity. A subset of TNBC is characterized by abundant levels of TILs accompanied by active inflammatory and interferon-related signaling, which facilitates the establishment of effective antitumor immune responses (41, 42). In contrast, other TNBCs exhibit stromal enrichment, chronic inflammation–associated T cell dysfunction, or locally suppressive metabolic conditions, resulting in immunosuppressive or immune-excluded phenotypes, in which immune responses may remain limited despite a high mutational burden (43). By comparison, HR^+^/HER2^-^ and a proportion of HER2^+^ tumors typically show low immune cell infiltration and more pronounced defects in antigen presentation, with TMEs that predominantly adopt immune-desert or immune-excluded architectures, thereby further reducing sensitivity to immunotherapy (44). Early-stage TNBC more frequently presents with elevated levels of TILs, and increased TILs are closely associated with improved immunotherapy responses and more favorable long-term outcomes. In parallel, TNBC is more likely to exhibit increased PD-L1 expression together with higher TMB, and shows enrichment of immune-related signaling pathways at the molecular level (45, 46).

Among these immunological features, TILs represent one of the most extensively studied biomarkers and have been shown to have important prognostic and predictive value based on extensive clinical and translational evidence. In particular, in TNBC, high levels of TILs generally reflect a local immune environment that is more permissive to antitumor immune responses and represent one of the most robust indicators of immunotherapy sensitivity (41). In studies of immunotherapy for metastatic TNBC, clear efficacy associations have also been observed. Correlative analyses from the KEYNOTE-086 trial showed that higher stromal levels of TILs were associated with increased objective response rates. This trend was more evident in the first-line setting and among PD-L1–positive patients (47). The breast cancer TME is dynamic, and its immune state can be remodeled under therapeutic interventions. Approaches that improve antigen presentation, adjust the balance of immunosuppressive cell populations, reprogram macrophage function, or alleviate stromal, metabolic, and hypoxic stress may enhance local immune activity (48). Neoadjuvant immunotherapy studies have further provided clinical evidence for dynamic TME remodeling during treatment. Studies exemplified by KEYNOTE-522 showed that increased tumor immune activity after treatment was closely associated with lower residual cancer burden (RCB), suggesting that local immune activation is clinically relevant to therapeutic benefit (49). Meanwhile, biomarker analyses from the GeparNuevo trial indicated that baseline stromal levels of TILs and on-treatment increases in intratumoral TILs were associated with pCR outcomes. This association was not observed in chemotherapy-only cohorts, supporting the consistency between immunotherapy-associated local immune changes and efficacy (50). Based on these insights, research efforts are increasingly expanding from exclusive reliance on systemic administration to more spatially targeted local immunomodulatory strategies, including intratumoral delivery systems, microenvironment-responsive nanoplatforms, and DMNs as emerging localized delivery technologies (51, 52). Collectively, the high plasticity of the breast cancer TME highlights the potential of local delivery approaches to enhance antigen presentation, promote effector T cell recruitment, and attenuate immunosuppressive networks, thereby providing a feasible strategy for improving the efficacy and safety of breast cancer immunotherapy.

## 3 Structural basis and delivery characteristics of dissolving microneedle systems

### 3.1 Material composition and microstructural design

With the increasing emphasis on localized delivery strategies in cancer immunotherapy, MNs have gradually emerged as an important technological platform for overcoming the skin barrier (53). Based on material properties and delivery modalities, MNs can be classified into solid, coated, hollow, dissolving, and hydrogel-forming types. Among these, solid and coated MNs are more suitable for enhancing transdermal permeability or enabling rapid release of small quantities of formulations, whereas hollow MNs are primarily designed for the local delivery of larger doses or sustained administration (54). With advances in polymer processing technologies, DMNs have gained particular attention. DMNs encapsulate therapeutic agents within biodegradable polymer matrices and release their payload upon dissolution after penetrating the stratum corneum. This design confers favorable biocompatibility, flexible drug loading, and improved safety by avoiding needle residue. These features make them especially suitable for the delivery of bioactive molecules such as antigens, nucleic acid adjuvants, and immune agonists (13). During the design and fabrication of DMNs, both the curing process and the selection of matrix materials directly influence the stability of encapsulated agents and the overall performance of the microneedles. An ideal matrix polymer should provide sufficient mechanical strength and good biocompatibility while protecting the encapsulated therapeutics without compromising their safety or biological activity. Moreover, the drug release behavior of DMNs is jointly regulated by material properties and structural design, and is typically governed by a combination of drug diffusion, matrix dissolution, and degradation processes (55). Therefore, rational selection of matrix polymers represents a critical prerequisite for achieving efficient and controllable DMN-based delivery. DMNs are commonly fabricated from hydrophilic polymers such as hyaluronic acid (HA), polyvinyl alcohol (PVA), polyvinylpyrrolidone (PVP), chitosan (CS), carboxymethyl cellulose (CMC), chondroitin sulfate (ChS), and silk fibroin (56–59). By modulating polymer molecular weight, crosslinking density, and blending ratios, or by combining different polymers, dissolution behavior can be precisely regulated while maintaining adequate mechanical strength (**Table 1**). Such tunable design enables DMNs to meet the requirements for efficient, stable, and precisely controlled local delivery in breast cancer immunotherapy (59, 60). In addition, some studies have explored DMN structures composed entirely of therapeutic molecules themselves, allowing higher local drug loading densities while minimizing the introduction of excipients, thereby offering new material design strategies for potent immune activation (61).

**Table 1:** Material systems, structural design, and key characteristics of dissolving microneedles in immunodelivery.

Material	Structure and fabrication	Immunodelivery mechanism	Limitations	References
PVP (polyvinylpyrrolidone)	Micromolding fabrication; homogeneous needle matrix with drugs dispersed throughout	Rapid dissolution, generating transient high local concentrations at the epidermal–dermal junction, thereby inducing early activation of cutaneous APCs	Limited mechanical strength; excessively rapid release, unfavorable for sustaining immune responses	(71)
PVA (polyvinyl alcohol)	Micromolding combined with vacuum filling; tunable crystallinity with favorable mechanical properties	Gradual hydration and dissolution, prolonging antigen exposure at local immune sites and supporting sustained DC uptake and enhanced initial T cell activation	Limited long-term storage stability; relatively high demand for repeated administration	(72)
HA (hyaluronic acid)	Natural polysaccharide matrix; micromolding or two-step fabrication; drug enrichment at the needle tips	Rapid dissolution in interstitial fluid, with drug deposition in the superficial dermis, promoting APC uptake and migration toward draining lymph nodes	Sensitive to humidity; formulation stability and scalability remain challenging	(73, 74)
CMC (carboxymethyl cellulose)	Two-step casting; dense arrays with controllable swelling behavior	Swelling-induced formation of stable microchannels, enabling diffusion-driven sustained release, maintaining local immune stimulation and supporting continuous DC activation	Limited drug loading capacity	(75)
Chitosan	Micromolding combined with chemical crosslinking; positively charged needles	Positive charge enhances local antigen retention and APC interactions in the skin, while activating innate immune signaling and amplifying adaptive immune responses	Insufficient mechanical strength; limited scalability	(76)
PVA/PVP	Micromolding; tip–base compartmentalized structure	Rapid dissolution of needle tips for immune priming, with sustained release from the base to maintain stimulation, resulting in staged immune input	Prolonged treatment duration; multiple administrations required	(77, 78)
PVA/PLGA	Thermal molding or layered casting; core–shell or multilayer structures	Rapid release from the soluble outer layer to initiate immune activation, followed by sustained release from the PLGA core to maintain stimulation, coordinating immune priming and effector phases	Higher production costs; uncertainty in clinical translation	(79)
Carrier-free drug/protein microneedles	Direct mold fabrication; needles composed entirely of active agents	Direct dissolution generates high local concentrations of antigens or immunomodulators at cutaneous immune sites, minimizing excipient interference and enhancing immune activation	Increased needle brittleness; stringent storage and handling requirements	(69)

**Figure 1:**
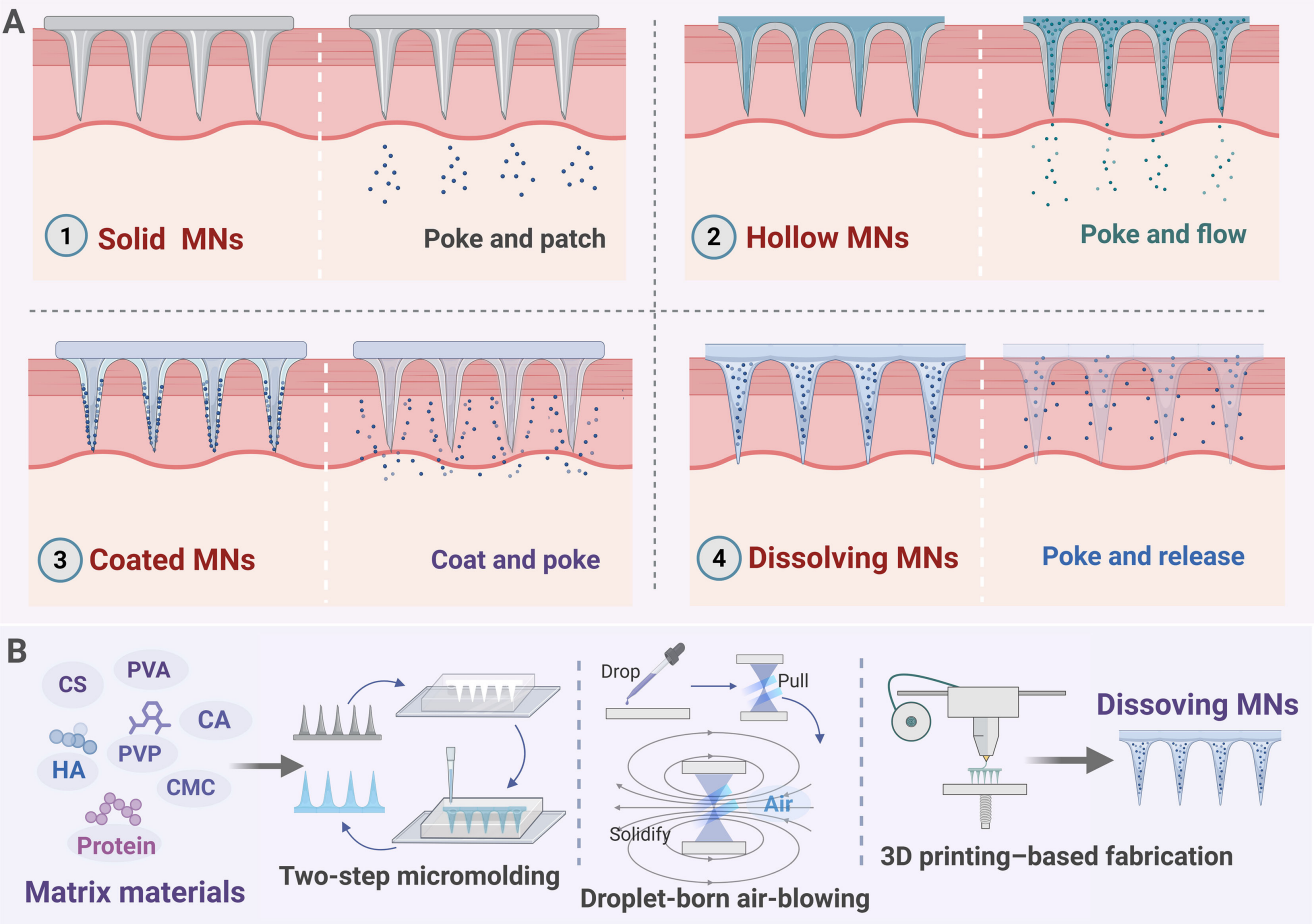
Transdermal delivery modes of microneedle platforms and an overview of dissolving microneedle fabrication routes. **(A)** Schematic illustration of representative delivery mechanisms. Solid microneedles create microchannels that enable subsequent diffusion-based transport (“poke and patch”); hollow microneedles deliver liquid formulations through a central lumen (“poke and flow”); coated microneedles deposit surface-loaded cargos upon insertion (“coat and poke”); and dissolving microneedles (DMNs), fabricated from soluble or biodegradable matrices, release payloads upon hydration-driven dissolution in the skin (“poke and release”). **(B)** Overview of matrix material categories and representative fabrication approaches for DMNs, including two-step micromolding, droplet-born air-blowing, and 3D printing, which enable controllable needle formation and payload distribution. Created with BioRender.com.

From a structural design perspective, DMNs can be engineered with needle lengths ranging from several tens to several thousand micrometers. In practical immunodelivery applications, needle lengths of 300–800 μm are most commonly employed to ensure effective penetration of the stratum corneum while avoiding damage to deeper tissues. Geometric parameters such as tip radius, needle taper, and array density directly influence insertion efficiency, intradermal drug deposition, and local diffusion patterns (62). With fabrication techniques evolving from conventional polymer molding, centrifugal casting, and vacuum filling toward three-dimensional printing and photopolymerization processes, improvements in microneedle shape uniformity and manufacturing reproducibility have been achieved, providing a more reliable structural basis for stable loading of complex biologics (63) (**Figure 1**). Further refinement through hierarchical and spatial design strategies, including tip-loaded or core–shell architectures, enables spatial segregation of active components within different regions of the microneedle, thereby allowing delayed or multistage release profiles that better align with the temporal requirements of antigen presentation and immune stimulation (64, 65). Building on these designs, integration of delivery carriers such as nanoparticles, liposomes, or polymeric microcapsules into DMN matrices can enhance the stability of sensitive formulations, including proteins and nucleic acids, without compromising insertion performance. This approach creates a dual-layer “nanocarrier–microneedle” delivery system within the skin, offering greater structural and functional flexibility for multistage immune modulation (66). Furthermore, recent studies have demonstrated that such multilayered or composite dissolving structures not only prolong the residence time of immunotherapeutics in the skin but also sustain local immune stimulation, thereby enhancing the overall efficacy of transdermal immunomodulation (67, 68). In addition, cryomicroneedles, as an emerging variant of DMNs, can preserve the biological activity of protein-based and live-cell formulations during fabrication and storage, showing potential value for cell-based immunotherapy delivery. However, their dependence on cold-chain conditions also presents challenges for clinical translation (69, 70). Overall, the coordinated optimization of material systems and microstructural design enables DMNs to meet requirements for insertion, safety, and stability. It also provides the capability to modulate delivery processes and biological effects.

### 3.2 Advantages of transdermal immunodelivery

As the largest immunologically active interface of the body, the skin is enriched with Langerhans cells, dermal dendritic cell subsets, and multiple classes of innate lymphoid cells. This highly organized immune architecture provides a unique and efficient biological foundation for *in situ* immune priming. In this context, DMNs offer a minimally invasive and controllable transdermal delivery strategy for immunotherapeutic agents. DMNs traverse the stratum corneum barrier and enable the precise delivery of antigens, immune adjuvants, and immunomodulatory molecules into the epidermis and superficial dermis. This allows extensive interaction with cutaneous immune cell networks prior to systemic circulation. This localized exposure establishes a highly concentrated immunostimulatory microenvironment in the skin, supporting efficient and repeatable cutaneous immune modulation (80, 81). Studies using ovalbumin (OVA) as a model antigen have demonstrated that transdermal co-delivery of antigens with adjuvants such as poly(I:C) via DMNs generates high-density antigen exposure in superficial skin layers. This approach markedly enhances antigen-specific cellular and humoral immune responses, with response magnitudes exceeding those achieved by conventional intramuscular injection (82). In addition, encapsulation of antigens within poly(lactic-co-glycolic acid) (PLGA) nanoparticles followed by DMN-mediated delivery facilitates efficient transport of particulate antigens by skin-derived DCs to draining lymph nodes. This process enhances antigen cross-presentation and amplifies antigen-specific immune responses, while nanoparticle encapsulation further improves antigen stability and prolongs intradermal retention, thereby supporting sustained and stable immune stimulation (83). Experimental evidence indicates that antigens delivered via microneedles are more readily captured by specific skin DC subsets. These cells exhibit a pronounced capacity to migrate to draining lymph nodes and induce CD8^+^ T cell responses. This feature effectively strengthens cytotoxic immune responses and is of particular relevance for breast cancer therapies that rely on T cell–mediated immunity (84, 85).

Furthermore, transdermal delivery avoids rapid dilution of therapeutics in the systemic circulation, allowing the formation of a locally retained delivery environment within the skin that sustains immune activation signals over extended periods. This prolonged exposure promotes DC maturation, enhances inflammatory cytokine expression, and improves the efficiency of effector T cell priming (86–88). Moreover, by substantially reducing systemic drug exposure, transdermal immunodelivery can lower the risk of immune-related adverse events while preserving or enhancing immunostimulatory efficacy, a property of particular clinical relevance for breast cancer immunotherapy regimens that incorporate potent immune adjuvants (89). During amplification of antitumor immune responses, transdermal delivery can circumvent immunosuppressive structures within the tumor itself. It enables immune activation to occur initially in the skin and its draining lymph nodes, followed by migration of expanded effector T cells to tumor sites. This sequence enhances tumor infiltration and strengthens antitumor immune activity (67). Accumulating evidence suggests that this “peripheral priming–systemic dissemination” mode of immune activation is compatible with the controllable release characteristics achieved by DMNs in the skin and has demonstrated superior antitumor immune efficacy compared with conventional delivery routes across multiple tumor models (90). In addition, the minimally invasive nature and procedural repeatability of DMNs support their feasibility for repeated administration or long-term immune modulation, thereby improving practical implementation and patient adherence (91). Overall, transdermal immunodelivery strategies enable localized and controllable immune signal input at the cutaneous immune interface. This enhances the spatial precision of immune priming while reducing systemic exposure risks, thereby supporting the establishment and maintenance of stable antitumor immune states.

## 4 Applications of dissolving microneedles in breast cancer immunotherapy

### 4.1 Microneedle-based delivery of tumor vaccines

During the development of breast cancer immunotherapy, therapeutic tumor vaccines have been widely regarded as an important strategy to address insufficient antigen presentation and immunologically “cold” tumor microenvironments. However, conventional subcutaneous or intramuscular injection routes often fail to achieve efficient antigen delivery and early immune priming in breast cancer, while adjuvant-associated systemic toxicity further constrains vaccine efficacy. As a delivery modality targeting the cutaneous immune interface, DMNs are driving the evolution of tumor vaccines from simple formulations to platform-based systems with intrinsic immunomodulatory capacity. The skin is enriched with Langerhans cells and dermal DCs and possesses a structural advantage for efficient antigen transport to draining lymph nodes. Consequently, antigens delivered via DMNs can establish high-density exposure within superficial skin layers prior to systemic circulation, thereby markedly enhancing DC uptake, processing, and migratory efficiency. Accumulating evidence indicates that this skin-initiated mode of immune priming effectively alleviates insufficient immune activation in breast cancer, synchronizes early T cell activation, and improves the overall quality of immune responses (92, 93).

In practical immunodelivery applications, DMNs accommodate a broad spectrum of vaccine formats, including peptide antigens, fusion proteins, multi-epitope antigens, nucleic acid vaccines, and vaccines based on cell membranes or whole-cell lysates. They consistently demonstrate superior immune amplification compared with conventional injection routes in breast cancer models. For example, in the metastatic 4T1 breast cancer model, DMN-mediated delivery of whole-cell lysate antigens significantly enhanced DC uptake and migration to draining lymph nodes, increased the proportion of functional CD8^+^ T cells and reduced pulmonary metastasis, indicating enhanced immune responses across priming and effector phases (94, 95). Studies on multi-epitope antigens show that transdermal delivery maintains greater T cell clonal diversity and sustains effector T cell function within the TME (96). In the context of nucleic acid vaccines, microneedle-mediated localized transfection and *in situ* antigen expression markedly augment CD8^+^ T cell responses and improve presentation efficiency for low-abundance antigens (97). With advances in materials science and nanoimmunoengineering, nanocarrier–microneedle co-delivery strategies have further reinforced the immunogenicity and antitumor efficacy of tumor vaccines (98). For breast cancer subtypes with pronounced immune heterogeneity, such as TNBC, increasing efforts focus on integrating multi-antigen or multicomponent systems into microneedle structures. These include tumor cell membrane nanoparticles, exosomes, and antigen nanocomplexes to expand antigen coverage and enhance cross-presentation at cutaneous immune sites (99–101). Gheybi et al. demonstrated in BALB/c mice bearing 4T1 tumors that chitosan nanoparticle–based microneedles loaded with recombinant CD44v antigens effectively induced antigen-specific immune responses and suppressed tumor growth (102). Similarly, rNap chitosan nanoparticle microneedle vaccines developed by Mohabati Mobarez et al. exhibited enhanced immune activation and inhibition of tumor progression in both 4T1 models and MCF-7 systems (103). Microneedle systems incorporating PLGA- or chitosan-based nanoparticles loaded with breast cancer–associated antigens, such as MAM-A or CD44v, further improve antigen targeting and immune activation efficiency. For instance, chitosan nanoparticle microneedles carrying MAM-A antigens significantly enhanced immune responses and suppressed tumor progression in DMBA- and MNU-induced breast cancer mouse models (104). In addition, delivery of tumor cell lysates loaded into mannose-modified chitosan nanoparticles via microneedles markedly enhanced DC uptake and antitumor immune responses in melanoma models, providing immunological rationale for mechanistic translation to breast cancer settings (105).Further studies combining mannose-modified chitosan nanovaccines with BCG polysaccharide nucleic acid (BCG-PSN) adjuvant in DMN patches demonstrated enhanced DC maturation in tumor-draining lymph nodes and inhibition of 4T1 tumor growth without evident systemic toxicity (106). Collectively, advances in materials–immunology integrated design are driving breast cancer vaccines from conventional antigen carriers toward platform-based and tunable immunomodulatory systems. This provides a critical foundation for achieving stable and predictable antitumor immune responses.

### 4.2 Microneedle-based delivery of immune agonists

Within DMN-based cancer immunotherapy, localized delivery of immune agonists has emerged as an important strategy for overcoming the immunosuppressive microenvironment characteristic of breast cancer. Unlike tumor vaccines, which primarily aim to broaden antigen repertoires and initiate primary immune responses, immune agonists mainly function by activating innate immune sensing, promoting DC maturation, and amplifying inflammatory signaling. These effects alleviate the suppression of effector T cells by immunosuppressive cell populations, such as MDSCs and Tregs. In breast cancer models, dissolving polymer–based microneedle structures enable the formation of high local drug concentrations within the dermis. This facilitates type I interferon cascades and antigen cross-presentation, thereby enhancing CD8^+^ T cell recruitment and tumor infiltration. This kinetic pattern of “peripheral immune priming followed by intratumoral effector amplification” offers greater spatial precision and safety than systemic administration (107). Building on this concept, several studies have integrated nanodelivery systems into DMNs to improve local immunomodulatory efficiency. Huang et al. reported a localized immunomodulatory strategy based on DMNs incorporating nanocarriers. In TNBC models, co-delivery of the TLR7/8 agonist resiquimod (R848) and anti–PD-1 antibody enhanced antigen presentation, expanded tumor-infiltrating effector CD8^+^ T cells, and promoted immune memory–associated responses. This markedly improved the immunosuppressive TME and therapeutic efficacy (16). In addition, by combining immune agonist strategies with small molecules that induce immunogenic cell death or ferroptosis, microneedle-based delivery can simultaneously enhance tumor cell immunogenicity and antitumor immune responses, while establishing sustained immunostimulatory conditions at postoperative local sites, thereby improving local immune defense in patients at high risk of recurrence (108). Recent studies show that HA-based DMNs can locally co-deliver ferroptosis-inducing nanomaterials and sorafenib. This approach significantly suppresses tumor growth in TNBC models while reducing systemic toxicity, suggesting that DMNs serve as effective delivery platforms for inducing immunogenic cell death and amplifying local immune activation (109). On the basis of such combinatorial concepts, shell–core structured DMNs have been developed. The outer layer rapidly releases doxorubicin (DOX) to induce immunogenic cell death and promote tumor antigen release, while the inner core provides sustained release of carrier-free nano-adjuvants to capture antigens *in situ* and amplify innate immune signaling. This design enhances antitumor immune responses and further improves therapeutic efficacy when combined with anti–PD-1 therapy (66). In addition, integration of the small-molecule targeted agent neratinib into DMN systems via nanocarriers enables sustained and controllable local drug exposure in breast cancer lesions while reducing systemic toxicity, providing a feasible delivery basis for combination with immune agonists or immunotherapeutic strategies (110). With the rapid convergence of materials science and systems engineering, emerging wearable, stimuli-responsive, or externally assisted microneedle architectures further enhance the spatial enrichment and biological activity of immunomodulatory agents within the skin, exhibiting stronger immune activation and antitumor effects than conventional injection routes across multiple solid tumor models (111, 112). These trends indicate that localized delivery strategies centered on the cutaneous immune axis are becoming an important direction for focal immune reconstruction in breast cancer.

In recent years, DMN-based immune agonist delivery has evolved from single-agent carriers to immunomodulatory systems capable of multi-pathway intervention. Designs incorporating responsive hydrogels, multilayer architectures, or thermally regulated release allow agonists to be delivered in temporally and spatially staged manners within the skin, thereby better matching the sequential requirements of innate immune activation and adaptive immune expansion (113, 114). Meanwhile, integration of immune agonists with physical or chemical interventions, such as photothermal therapy, gas therapy, or metal ions, into DMN structures can induce local release of danger-associated molecular patterns and immunogenic cell death, further expanding DC recruitment and functional T cell expansion (115). Incorporation of stimulator of interferon genes (STING) pathway agonists, such as Mn²^+^, into DMNs locally amplifies innate immune signaling at cutaneous immune sites, enhances DC activation and CD8^+^ T cell responses, and significantly increases sensitivity to immunotherapy in breast cancer models (116). In addition, recent studies have explored DMN-mediated local delivery of CRISPR/Cas gene-editing systems to attenuate tumor-associated immunosuppressive signaling at the genetic level, thereby enhancing effector CD8^+^ T cell responses while reducing systemic off-target risks, highlighting the potential of DMNs as upstream regulatory tools for immune agonist–based strategies (117). In some studies, dissolving or hydrogel-forming microneedles have been further engineered as self-regulating or self-amplifying immunomodulatory systems, in which drug release and metabolic modulation are triggered by TME-associated cues or external physical stimuli, enabling dynamic regulation of immunosuppressive TMEs (118). Although early investigations primarily focused on cutaneous tumors such as melanoma, recent work targeting breast cancer—particularly TNBC and postoperative high-risk recurrence subgroups—has expanded substantially. Multiple DMN-based systems incorporating STING agonists, ferroptosis inducers, or locally enhanced radiotherapy have now been validated in breast cancer models, underscoring the clear translational potential of DMNs in this context (119). Overall, microneedle-mediated delivery of immune agonists enables efficient and controllable local immune activation at cutaneous immune sites. It effectively alleviates key limitations in breast cancer, including insufficient immune priming, accumulation of immunosuppressive cell populations, and restricted effector T cell function, and provides a spatially precise and programmable pathway for focal immune reconstruction.

### 4.3 Microneedle-based delivery of immune checkpoint inhibitors

The central limitation of breast cancer immunotherapy remains the persistent state of T cell exhaustion within tumors, together with the immunosuppressive network sustained by the PD-1/PD-L1 axis. This condition not only compromises the cytotoxic function of effector T cells but also impedes the reinitiation of antigen presentation and subsequent immune cycling (120). Although ICIs have demonstrated clinical benefit in both neoadjuvant and metastatic TNBC settings, their efficacy depends on achieving sufficient and sustained exposure at tumor-relevant immune sites. Conventional administration routes often fail to maintain adequate local blockade within the tumor microenvironment. They are also frequently accompanied by irAEs, which substantially constrain the therapeutic window (121). In parallel, aberrant tumor vasculature, impaired lymphatic drainage, and stromal barriers further limit antibody penetration into tumor cores, reducing the efficiency of local PD-1 blockade and facilitating immune tolerance. Given the persistent immunosuppressive state in breast cancer and the spatial constraints of ICIs, localized delivery strategies have been proposed to enhance checkpoint inhibition at critical immune sites. By optimizing the spatial context of immune activation and strengthening local blockade, such approaches offer new avenues for mitigating tumor-associated immune suppression. DMNs enable localized delivery at the skin, an immune-enriched interface. This allows PD-1 blockade to preferentially engage dendritic cells and early T cell activation processes. This reinforces peripheral immune priming and promotes effector T cell infiltration into tumor tissues, thereby improving both local and distant tumor control (122).

From an immunoregulatory perspective, the therapeutic outcome of ICIs is determined not only by blockade intensity. It also depends on their spatial exposure and temporal coordination during immune priming and effector expansion. The localized immune activation pattern established by transdermal delivery provides favorable conditions for optimizing PD-1/PD-L1 blockade across different stages of the immune response. For example, stimuli-responsive microneedle systems can achieve on-demand release of PD-1 inhibitors within the skin, significantly enhancing CD8^+^ T cell infiltration and suppressing tumor growth, thereby validating the feasibility of initiating checkpoint blockade at peripheral immune sites (123). In addition, microneedle platforms based on natural polysaccharides such as hyaluronic acid can amplify local innate immune signaling through CD44- or TLR-mediated signaling pathways, further synergizing with checkpoint inhibition (124). Evidence suggests that temporal coordination of immune stimulation and PD-1 blockade exposure can promote dendritic cell maturation and effector T cell expansion while reducing the risk of early T cell exhaustion (70). Cryogenic DMNs co-delivering dendritic cell vaccines and PD-1 inhibitors have been shown to simultaneously fulfill antigen delivery and checkpoint blockade functions, inducing systemic immune amplification and effectively suppressing distant metastatic lesions. Within stimuli-responsive systems, photothermal microneedles enhance tumor antigen release by inducing immunogenic cell death (ICD), thereby amplifying PD-1 blockade–mediated effector T cell expansion. For instance, dissolving microneedle patches loaded with gold nanorod photothermal agents and anti–PD-1 peptides induced photothermal ICD upon near-infrared irradiation in 4T1 breast cancer models. This synergized with PD-1 blockade to enhance CD8^+^ T cell–mediated antitumor responses while exhibiting lower systemic toxicity than conventional administration routes (125). Moreover, the STING pathway has emerged as a suitable target for localized microneedle delivery. Previous studies have demonstrated that microneedle-mediated delivery of STING agonists can sustain type I interferon signaling locally, enhance antigen presentation, and improve effector T cell recruitment, thereby significantly increasing tumor responsiveness to PD-1 blockade (126). To further expand the spatial coverage of local immune modulation, ultrasound-assisted microneedle delivery strategies have gained increasing attention. Wearable flexible ultrasound patches can substantially enhance transdermal drug flux via sonoporation and cavitation effects, while ultrasound-responsive nanoparticles encapsulated within DMNs, combined with cavitation ultrasound exposure, further improve intratumoral penetration depth and antitumor efficacy in superficial solid tumor models. Such approaches provide regionally targeted immunotherapeutic options with translational potential for postoperative chest wall lesions and locoregional recurrence in breast cancer (127, 128). Collectively, these studies indicate that microneedle-mediated immunotherapy delivery is evolving from a strategy that merely alters administration routes into a localized immunomodulatory platform capable of coordinating antigen presentation, effector T cell expansion, and reversal of immunosuppression, thereby underscoring its substantial potential in breast cancer immunotherapy (**Table 2**).

**Table 2:** Microneedle-mediated delivery strategies in breast cancer immunotherapy and their effects on tumor microenvironment remodeling.

Delivery strategy	Payloads	Experimental models	Major immunological effects and TME remodeling	Translational implications	References
Tumor vaccine delivery	Tumor-derived total RNA, multi-epitope antigens, tumor cell membranes or lysates	4T1, EMT6 and other murine breast cancer models	Enhances maturation and migration of skin and draining lymph node DCs; improves antigen uptake and cross-presentation efficiency; promotes expansion and tumor infiltration of antigen-specific CD8^+^ T cells; improves the CD8^+^/Tregs balance and partially reverses immunosuppressive TME	Improves limited immune priming and extends coverage to low-immunogenic breast cancer subtypes; constrained by antigen standardization and formulation stability	(94, 95, 103, 129)
Immune agonist delivery	TLR7/8 agonists, STING pathway activators	Orthotopic and metastatic TNBC models	Amplifies innate immune sensing and type I interferon signaling; promotes DC activation; enhances intratumoral CD8^+^ T cell recruitment and expansion while reducing Tregs and MDSCs accumulation, thereby remodeling immunosuppressive TME	Enhances immunotherapy sensitivity while reducing systemic exposure; requires careful control of local immune activation intensity	(16, 116, 122, 130)
Immune checkpoint blockade delivery	Anti–PD-1 or anti–PD-L1 antibodies	Orthotopic and distant TNBC tumor models	Alleviates intratumoral T cell functional exhaustion and enhances effector T cell infiltration; improves IFN-γ–associated signaling pathways and supports maintenance of effector immunity while weakening suppressive immune networks	Reduces immune-related adverse events and supports repeated or long-term dosing; limited by antibody loading capacity and cost	(107, 123, 124)
Immunogenicity-enhancing delivery	Chemotherapeutic agents, ferroptosis inducers, or photothermal materials	Orthotopic tumors and postoperative residual disease models	Induces immunogenic cell death and promotes release of tumor antigens and DAMPs; enhances DC cross-presentation and amplifies CD8^+^ T cell responses, remodeling a locally inflamed TME	Applicable for postoperative recurrence and metastasis risk control; requires balancing local tissue tolerance	(108, 131, 132)
Physically assisted immunodelivery	Ultrasound, photothermal or piezoelectric materials	Orthotopic and locally recurrent TNBC models	Improves local tissue permeability and synergistically activates innate immune signaling; promotes immune cell recruitment and reshapes suppressive myeloid cell composition, amplifying local immune responses	Significantly enhances local immune efficacy; translation depends on device integration and procedural standardization	(111)
Postoperative local immunodelivery	Immune agonists, immunogenicity-enhancing components, or immunomodulatory molecules	Peri-incisional or tumor bed models	Maintains postoperative local immune activity and promotes formation of tissue-resident memory T cells; restricts re-establishment of immunosuppressive cell populations and enhances local immune surveillance	Well suited for defined postoperative clinical scenarios with high patient compliance; long-term safety requires further validation	(118, 133, 134)

## 5 Mechanisms of immune microenvironment remodeling mediated by dissolving microneedles

### 5.1 *In situ* mechanisms of skin immune priming

In DMN-mediated breast cancer immunotherapy, the skin constitutes the primary interface at which immune responses are first initiated. The skin exhibits a pronounced stratified architecture and cellular heterogeneity, within which Langerhans cells (LCs) and multiple subsets of dermal DCs together form a critical peripheral antigen-presenting network that cooperatively participates in exogenous antigen capture, processing, and migration to draining lymph nodes (43, 135). Substantial evidence indicates that the migratory capacity and functional state of LCs and dermal DCs are highly plastic and can be dynamically regulated under steady-state and inflammatory conditions. This enables the cutaneous immune system to generate hierarchical and remodelable antigen-presenting responses to transdermal stimulation (136). Mild micro-injury induced by DMN penetration of the stratum corneum triggers keratinocytes and skin-resident immune cells to release proinflammatory mediators such as IL-1 and TNF-α, along with the generation of multiple damage-associated molecular patterns (DAMPs). These signals collectively drive skin DCs to transition from a steady-state toward a mature phenotype and promote their migration to draining lymph nodes through upregulation of CCR7 (137). Meanwhile, microneedles create localized antigen-enriched regions at the epidermal–dermal junction, allowing antigen uptake by DCs residing at different skin layers to occur in a more efficient and synchronized manner. As DMNs gradually dissolve within the skin, delivered antigens and immunological adjuvants remain continuously exposed at local immune sites. This provides sustained support for antigen uptake, functional maturation, and migration of DCs to draining lymph nodes (138). For example, tumor cell membrane antigens integrated with the TLR7 agonist R837 into membrane-coated nanoparticles and loaded into DMNs were shown to enhance antigen uptake by skin-resident APCs and upregulate immune mediators such as IFN-γ and TNF-α following transdermal delivery, resulting in effective antitumor immune output (139). When antigen-loaded mature DCs enter lymphatic vessels and reach draining lymph nodes, immune responses initiated in the skin extend into the adaptive immune phase. This provides essential priming signals for subsequent T-cell clonal expansion and functional differentiation (140). This mechanism has been further validated in studies in which tumor-derived total RNA and CpG adjuvants were formulated into nanovaccines and encapsulated within DMNs; transdermal delivery markedly enhanced antigen uptake and functional maturation of DCs in both the skin and tumor-draining lymph nodes, thereby amplifying antigen presentation efficiency and promoting CD8^+^ T-cell responses, highlighting the coordinated role of cutaneous immune sites and draining lymph nodes in transdermal immune regulation (95). Taken together, on the basis of antigen presentation established during transdermal immune priming, effective activation and functional engagement of naïve T cells represent a prerequisite for sustained immune expansion, a process that is already influenced by the local immune signaling milieu at early stages (141, 142).

Following *in situ* immune priming in the skin, migration of DCs to draining lymph nodes becomes a critical step in the subsequent propagation of immune responses. Within draining lymph nodes, mature conventional dendritic cell type 1 and type 2 (cDC1 and cDC2) subsets enhance antigen presentation and costimulatory signaling, broaden the TCR clonal repertoire, and promote the differentiation of CD8^+^ T cells toward cytotoxic effector phenotypes. In concert with key signaling pathways such as type I interferon responses, these processes establish a continuous cascade from localized priming to systemic immune amplification (143, 144). In contrast to single high-dose systemic administration, repeated transdermal dosing regimens are more favorable for maintaining T-cell functional stability, thereby reducing the risk of early exhaustion and supporting the gradual establishment of effector and memory T-cell populations (145). Notably, in breast cancer—particularly in immunologically heterogeneous subtypes such as TNBC—the long-term residency of intratumoral CD8^+^ T cells is closely associated with responsiveness to immunotherapy. Among these populations, tissue-resident memory T (T_RM) cells have attracted particular attention, as their spatial distribution and organizational relationships with tumor cells and vascular structures have been shown to correlate strongly with clinical outcomes (146, 147). Meanwhile, multiple studies suggest that the durable efficacy of immune checkpoint blockade depends not only on the abundance of intratumoral effector T cells, but also on the continuous supply of CD8^+^ T cells with progenitor or stem-like features from draining lymph nodes, which serve as a source for long-term maintenance of intratumoral T-cell populations. By contrast, excessive skewing of intratumoral CD8^+^ T cells toward terminal effector or tissue-resident phenotypes may compromise the ability of progenitor T cells to sustain ongoing immune responses (148). In this context, DMNs, by initiating immune responses in the skin and effectively linking draining lymph nodes with tumor sites, provide a biological basis for coordinating progenitor T-cell replenishment with intratumoral immune residency (149, 150).

### 5.2 Functional remodeling of tumor-infiltrating immune cells

In breast cancer, intratumoral immune responses often exhibit pronounced instability. Even when antigen-specific immune activation is achieved, effector immune responses often fail to be maintained over time. As a result, they are not effectively translated into durable tumor control. Particularly in TNBC, such immune instability arises more from functional and architectural constraints at the tissue level than from a simple lack of immune cell infiltration (151). Within the tumor microenvironment, hypoxia, metabolic competition, and immunosuppressive signaling coexist over prolonged periods. Under these conditions, the persistence of antitumor immunity depends on the functional plasticity of effector immune cells and the stability of antigen presentation. Accumulating evidence indicates that the efficiency and continuity of intratumoral antigen presentation are key limiting factors for the long-term maintenance of immune responses (152). In the local breast cancer milieu, cDC1 and cDC2 subsets sustain CD8^+^ T-cell effector function through continuous antigen presentation and cytokine support, thereby delaying the transition of these cells toward terminal differentiation or deep exhaustion (153). Effective infiltration of cytotoxic T lymphocytes (CTLs) into tumors is essential for antitumor immunity. Their recognition and killing of tumor cells through T-cell receptor (TCR)–major histocompatibility complex (MHC) interactions further enable the conversion of immune responses into stable tumor control. However, within immunosuppressive tumor microenvironments, stromal remodeling, metabolic stress, and inhibitory signaling networks often act in concert to compromise these processes (81). Accordingly, simply increasing immune cell infiltration is insufficient to ensure durable tumor control, as persistent interference with effector programs by suppressive microenvironmental factors remains a major bottleneck limiting immunotherapeutic efficacy (154).

**Figure 2:**
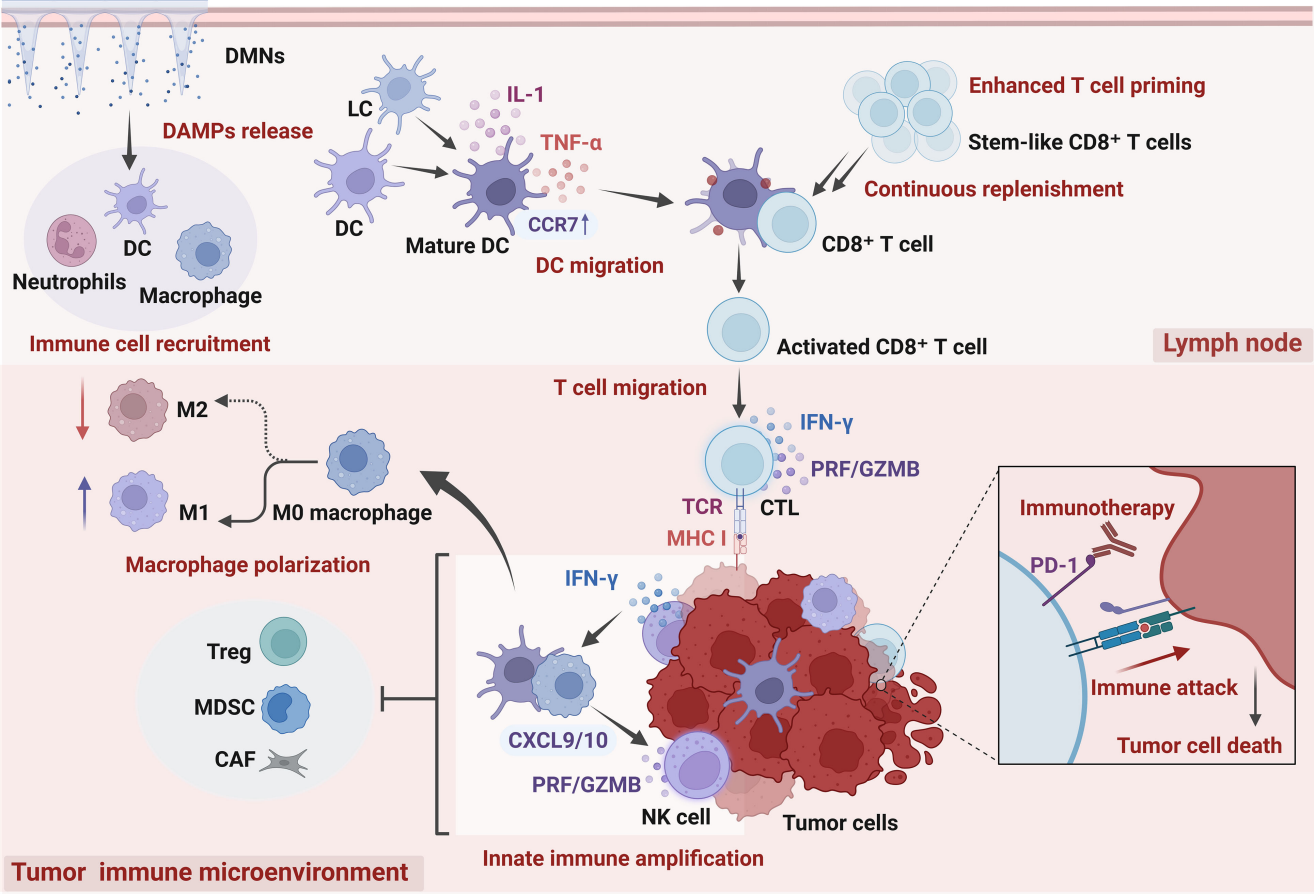
Schematic illustration of dissolving microneedle (DMN)–mediated peripheral immune priming and tumor immune microenvironment (TIME) remodeling. DMN insertion induces mild tissue injury and the release of damage-associated molecular patterns (DAMPs), promoting dendritic cell (DC) maturation and migration to draining lymph nodes, where CD8^+^ T-cell expansion occurs. Expanded CD8^+^ T cells infiltrate tumor tissues, differentiate into cytotoxic T lymphocytes (CTLs), and mediate tumor cell killing through IFN-γ and perforin/granzyme B (PRF/GZMB), together with innate immune cells such as natural killer (NK) cells. DMN-associated immune activation promotes macrophage polarization toward an M1-like phenotype, partially alleviates immunosuppression mediated by regulatory T cells (Tregs), myeloid-derived suppressor cells (MDSCs), and cancer-associated fibroblasts (CAFs), and may enhance PD-1/PD-L1 checkpoint blockade. Created with BioRender.com.

Compared with conventional systemic or intratumoral administration, DMNs establish a more stable immune input along the cutaneous immune axis, enabling antigen presentation and initial T-cell expansion to occur within a peripheral environment that is relatively less immunosuppressive. This spatial reprogramming favors the generation and maintenance of functionally intact effector T-cell populations that retain antitumor activity upon tumor infiltration (155–157). Nevertheless, long-term maintenance of intratumoral immune effects in breast cancer remains constrained by multiple immunosuppressive cell populations acting through convergent mechanisms. Tregs impose sustained suppression on intratumoral immune responses by weakening costimulatory signaling between APCs and effector T cells and limiting effector activation (158). MDSCs, in turn, impair the metabolic fitness and cytotoxic capacity of effector T cells within breast tumors through amino acid competition and accumulation of reactive oxygen and nitrogen intermediates (159). Immunosuppressive TAMs further contribute by reshaping the local cytokine milieu and maintaining inhibitory signaling networks, with their impact primarily reflecting functional reprogramming rather than simple numerical expansion (160). Experimental studies have demonstrated that selective depletion of MDSCs or Tregs can enhance antitumor immunity and suppress tumor progression in animal models, and that targeting tumor-infiltrating suppressive cells may reduce resistance to immune checkpoint blockade and amplify therapeutic efficacy (161, 162). However, direct depletion of suppressive immune cells remains limited in clinical settings by concerns regarding safety and selectivity. In this context, recent microneedle-based studies suggest that DMNs can function as localized immunomodulatory tools that indirectly alleviate innate immunosuppression and support adaptive immune maintenance by reshaping myeloid cell composition and functional states. For instance, integration of immunomodulatory polysaccharide matrices into dissolving microneedle platforms has been shown to induce a phenotypic shift of TAMs from immunosuppressive M2 states toward proinflammatory and phagocytic profiles, thereby enhancing intratumoral CD8^+^ T-cell infiltration and overall immune activation, indicating that DMNs can mitigate innate immune suppression through myeloid remodeling and support sustained adaptive immune responses (163). In the 4T1 breast cancer model, Xue et al. developed a wearable flexible ultrasound–dissolving microneedle patch in which ultrasound-activated piezoelectric nanoparticles locally generated reactive oxygen species, inducing immunogenic cell death and activating DCs. This approach promoted CD8^+^ T-cell infiltration and remodeled suppressive myeloid populations, and when combined with anti-PD-1 therapy, further amplified systemic antitumor immunity and established immune memory (111). Collectively, within this multifactorial suppressive context, peripheral immune input induced by DMNs does not directly replace intratumoral immune regulation. Instead, it indirectly influences the functional balance among tumor-infiltrating immune cells by continuously supplying effector T-cell populations with preserved functional potential. This creates more favorable conditions for sustained immune responses. Beyond suppressive populations, the functional states of CD4^+^ T-cell subsets and NK cells are likewise strongly shaped by the immune priming context, and their roles in cytokine support and innate immune amplification further determine the magnitude and durability of CD8^+^ T-cell responses (164, 165). Notably, once CD8^+^ T cells enter terminal differentiation or deep exhaustion within tumors, their capacity for functional restoration becomes markedly limited, thereby diminishing the therapeutic benefit of subsequent immune interventions (166). In breast cancer, particularly in TNBC, T-cell populations with tissue-resident features that retain functional plasticity are closely associated with immunotherapy responsiveness and long-term immune surveillance (167). On this basis, sustained peripheral immune priming via DMNs and the stable replenishment of functionally competent effector T cells may gradually reshape functional coordination among tumor-infiltrating immune cells without substantially increasing systemic toxicity (**Figure 2**). At the same time, the pronounced heterogeneity of breast cancer in terms of molecular subtypes, baseline immunogenicity, and tumor immune microenvironment composition may influence how DMN-mediated peripheral immune inputs operate across different tumor contexts and shape their immunological outcomes.

### 5.3 Immunological characteristics across molecular subtypes

Distinct molecular subtypes of breast cancer exhibit diverse immunological constraints during antitumor immune responses. These subtype-specific differences are closely associated with the heterogeneity of immunotherapy responsiveness and influence how localized immunodelivery strategies modulate immune responses across tumor contexts. TNBC generally displays higher baseline immunogenicity and antigen exposure; however, effector immune responses within tumors often lack persistence. Myeloid suppressive networks, metabolic competition, and stromal barriers rapidly reshape effector immune cell function after tumor entry, leading to functional attenuation and reduced durability of antitumor activity (168). Accordingly, in TNBC, the dominant immunological limitation is less related to defective immune priming and more concentrated at the level of effector-phase stability and persistence, which largely accounts for the pronounced heterogeneity in responses to immune checkpoint blockade observed in this subtype (169). In this context, transdermal immunodelivery strategies such as DMNs introduce antigens and immunostimulatory signals into the skin, a relatively organized immunoregulatory interface. This enables DC maturation, migration, and initial T-cell expansion to occur in a peripheral environment with weaker suppressive signaling, thereby establishing a more stable and functionally intact effector immune foundation at early stages (170). Across multiple breast cancer and TNBC preclinical models, microneedle-based immunointerventions have been shown to enhance the overall stability of effector immune responses after tumor entry, reducing susceptibility to early dysfunction within suppressive microenvironments and thereby sustaining continuous and effective antitumor immune activity. Further integration of immune agonists, immune checkpoint inhibitors, or immunogenic cell death–inducing strategies into DMN platforms can prolong localized immune activation without substantially increasing systemic toxicity. It also mitigates the disruptive effects of intratumoral suppressive structures on effector response continuity, thereby supporting more durable antitumor immunity (131).

By contrast, in HR^+^ and other immunologically “cold” breast cancer subtypes, the primary immunological constraints are predominantly manifested as insufficient immune priming and limited antigen presentation efficiency. The tumor microenvironment in these subtypes is characterized by inadequate effector T-cell recruitment, inefficient antigen presentation, and persistent immunosuppressive signaling, collectively hindering effective antitumor immune responses (171). Consequently, in HR^+^ and low-immunoreactivity settings, immunomodulatory strategies must prioritize overcoming defects in immune initiation and cell entry. Transdermal microneedle delivery enhances the utilization efficiency of antigens and immunomodulatory factors within the cutaneous immune network. This enables effective immune priming under low baseline immune activity and improves DC activation and initial T-cell expansion, thereby facilitating subsequent effector T-cell responses. For example, delivery of tumor-derived RNA nanovaccines via DMNs has been shown to markedly promote dendritic cell uptake and migration to draining lymph nodes in breast cancer models, resulting in enhanced CD8^+^ T-cell activation (172). Moreover, microneedle-mediated immune priming predominantly occurs in the skin and its draining lymph nodes rather than within the tumor itself, thereby partially decoupling immune initiation from the local immunosuppressive tumor microenvironment. This spatial separation helps reduce the direct interference of antigen presentation defects and persistent immunosuppression in HR^+^ tumors during the establishment of primary immune responses (173). Related studies further demonstrate that microneedle-based immunization strategies incorporating dendritic cell activation can significantly enhance antigen-presenting cell activation and T-cell responses, and that microneedle patches integrating immune agonists validate the feasibility of this immune priming pathway under low-immunoreactivity conditions in breast cancer models (174, 175). Taken together, the dominant constraints shaping antitumor immune responses differ substantially across breast cancer molecular subtypes, and the immunomodulatory effects of DMNs exhibit corresponding subtype-specific characteristics: in TNBC, their primary role lies in maintaining and amplifying existing effector immune responses, whereas in HR^+^ and immunologically “cold” subtypes, they predominantly function to alleviate impaired immune priming, enhance antigen presentation efficiency, and promote the establishment of initial effector T-cell responses (176).

## 6 Current applications and translational challenges

Although DMNs have demonstrated relatively robust immune amplification effects and the capacity to modulate the tumor immune microenvironment in preclinical studies of breast cancer immunotherapy, their clinical translation remains constrained by multiple practical challenges (177). At present, most studies are based on animal models and focus on enhancing immune priming through localized delivery to amplify systemic antitumor responses. As a result, issues related to population heterogeneity and representativeness remain insufficiently addressed, particularly in clinical settings. Unlike conventional injectable formulations, the immunological effects of DMNs depend primarily on the coordinated interplay among microneedle geometry, drug distribution, and intradermal release kinetics (55).Although microneedle structural parameters and release behaviors can be precisely engineered under laboratory conditions, they remain susceptible to variations in manufacturing, storage, and real-world administration. Such variability can lead to fluctuations in delivered dose and release profiles, which may ultimately translate into instability of immune outcomes (178, 179). Moreover, delivery performance observed in ex vivo skin models does not necessarily reflect *in vivo* efficacy. For example, dissolving microneedle arrays incorporating OVA/CpG-loaded PLGA nanoparticles achieved efficient skin penetration and rapid dissolution in ex vivo human skin. However, incomplete dissolution and reduced effective delivery doses were observed in mice, resulting in weaker antigen-specific T-cell responses compared with conventional intradermal injection of the same formulation. These findings indicate that fabrication processes and intraneedle drug distribution can influence effective dose delivery and release dynamics, thereby affecting the intensity and stability of immune input (180). Furthermore, much of the current evidence is derived from associative or observational findings in preclinical studies, and variations in study design and analysis, together with the limited availability of functional and mechanistic validation, may limit the robustness and causal interpretation of these findings. Consequently, the clinical feasibility of DMNs depends not only on delivery efficiency but also on consistent dosing and reproducible release kinetics across individuals and real-world conditions (52).

Meanwhile, the skin, as the delivery interface, exhibits pronounced structural and immunological heterogeneity, including differences in stratum corneum thickness, dermal density, local immune cell distribution, and baseline inflammatory status. In addition, variability in biological and clinical characteristics, such as disease stage, treatment background, and host conditions, may influence the consistency and interpretation of findings across different studies. These factors may increase variability in penetration depth, dissolution, drug release, and delivered dose, placing greater demands on delivery consistency and predictability (181). In parallel, breast cancer is characterized by substantial molecular and immunological heterogeneity, with distinct immune constraints across different molecular subtypes, making it unlikely that a single microneedle design can address all patient populations. Without clearly defined indications and application scenarios, the clinical value of DMNs risks being reduced to a mere alternative delivery route, thereby obscuring their immunomodulatory advantages. For immune agonists, antibodies, and nucleic acid–based therapeutics, therapeutic efficacy is highly sensitive to dose, release kinetics, and structural integrity; even minor variations in microneedle drug-loading capacity, spatial distribution, or stability may be magnified into significant differences in immune priming intensity or duration (182). Therefore, future studies should establish evaluation frameworks that better reflect underlying mechanisms, particularly by linking peripheral immune priming to intratumoral immune changes, to improve efficacy assessment and identify responsive populations. In addition, standardization of administration procedures and the development of corresponding quality control systems are required to improve reproducibility across patient populations and real-world use conditions. Finally, DMNs loaded with bioactive molecules face additional complexity with respect to sterilization strategies, quality assurance, and regulatory pathways, necessitating a translatable balance among delivery performance, preservation of biological activity, and immunological safety.

## 7 Conclusion

In recent years, with the growing recognition of the heterogeneity inherent to the tumor immune microenvironment of breast cancer, the focus of immunotherapy has gradually shifted from whether immune responses can be induced to whether they can be stably established and sustainably maintained. Within this context, DMNs enable the precise transdermal delivery of antigens and immunomodulatory signals into the skin, an immunologically active interface, thereby providing a localized strategy to influence both the anatomical site and the spatiotemporal dynamics of immune priming. Leveraging the high density of antigen-presenting cells in the skin, DMNs facilitate dendritic cell uptake and maturation, enhance the expansion of naïve T cells within draining lymph nodes, and provide a potentially more favorable immunological foundation for subsequent effector immune infiltration into tumor tissues. Through these mechanisms, they may help address the prevalent challenges of insufficient immune priming and immunosuppressive tumor microenvironments observed in breast cancer.

Accumulating evidence indicates that the therapeutic potential of DMNs in breast cancer immunotherapy extends beyond increasing local drug concentrations. It lies in their ability to initiate immune responses at peripheral sites, optimize antigen presentation, and support effector immune activation and amplification. On this basis, DMNs can be integrated with tumor vaccines, innate immune agonists, immunogenic cell death–inducing strategies, and immune checkpoint blockade in a complementary manner, thereby potentially enhancing the magnitude and durability of antitumor immune responses without markedly increasing systemic toxicity. However, the pronounced differences in baseline immunogenicity and dominant immune constraints across breast cancer molecular subtypes underscore the need for subtype-specific optimization and validation, guided by clearly defined beneficiary populations and clinical application scenarios. From a translational perspective, key challenges remain, including delivery consistency, formulation stability, indication delineation, and the predictability of immunological outcomes. Future efforts will require tighter integration across materials engineering, mechanistic immunology, and clinical trial design. This should be supported by standardized manufacturing and administration procedures, rational patient stratification, and evaluation frameworks that capture the relationship between peripheral immune priming and intratumoral immune remodeling. As our understanding of immune heterogeneity in breast cancer continues to advance, DMNs may represent a promising and potentially more precise and controllable immunomodulatory approach in selected molecular subtypes and clinical contexts, offering a novel avenue for localized immune intervention in breast cancer immunotherapy.
